# Changes in Hematological Parameters of Iron Status and Total Iron Concentrations in Different Biological Matrices during a Sports Season in Women’s Soccer Players

**DOI:** 10.3390/nu15081833

**Published:** 2023-04-11

**Authors:** Víctor Toro-Román, María C. Robles-Gil, Ignacio Bartolomé, Francisco J. Grijota, Diego Muñoz, Marcos Maynar-Mariño

**Affiliations:** 1 Faculty of Sport Sciences, University of Extremadura, Avenida de la Universidad s/n, 10003 Caceres, Spain; vtoro@unex.es (V.T.-R.);; 2 Department of Sport Science, Faculty of Education, Pontifical University of Salamanca, C/Henry Collet, 52-70, 37007 Salamanca, Spain

**Keywords:** mineral elements, football, nutrition, erythrocytes

## Abstract

Iron (Fe) metabolism and concentrations change during a sports season. Fe deficiency affects a significant number of women athletes. The aims of the present study were: (i) to analyze changes in hematological parameters of Fe status and (ii) to analyze changes in Fe concentrations in different biological matrices (serum, plasma, urine, erythrocytes, and platelets) during a sports season. Twenty-four Spanish semi-professional women’s soccer players (23.37 ± 3.95 years) participated in the present study. Three assessments were performed throughout the sports season (beginning, middle and end of the season). Nutritional intake was evaluated and female hormones, hematological parameters of Fe status and Fe concentrations in plasma, serum, urine, erythrocytes and platelets were determined. There were no differences in Fe intake. Hemoglobin and mean corpuscular hemoglobin concentrations increased at the end of the season compared to initial values (*p* < 0.05). There were no significant changes in extracellular Fe concentrations (plasma, serum, and urine). However, erythrocyte Fe concentrations were lower at the end of the season (*p* < 0.05). Hematological parameters of Fe status and intracellular Fe concentrations change throughout the sports season in women’s soccer players.

## 1. Introduction

Interest in women’s soccer has grown exponentially in recent years. The number of federated women players in 2019 was 13.3 million, and is estimated to increase to 60 million by 2026 [1]. The increase in the number of players is paralleled by an increase in the number of scientific publications on women’s soccer [2]. New research has made it possible to study the physical and physiological demands of women’s soccer players.

Regular physical training and soccer matches induce important physiological changes that should be evaluated. Monitoring these changes allows the coaching staff of soccer teams to adjust training loads [3]. A wide variety of physiological markers have been used for long-term monitoring of soccer players [4,5]. In particular, hematological markers have been considered important indicators of the body’s adaptation in response to different training loads [6]. Control of training intensity and volume could be responsible for changes in hematological values and iron (Fe) stores [7].

Fe deficiency is the most frequent nutritional deficiency [8,9], being more prevalent among women [8], and also affecting a large number of athletes [10,11,12]. Fe deficiencies have been reported in both individual [13,14] and team sports [8,15,16]. Athletes are at increased risk of Fe deficiency due to various mechanisms generated by physical training (hemolysis, Fe losses through sweating, gastrointestinal bleeding, etc.) [17]. Fe deficiency leads to impaired performance and early detection is crucial to prevent Fe deficiency and anemia [9].

Normally, Fe status is assessed indirectly by hematological parameters such as erythrocytes, hemoglobin, hematocrit, ferritin, and transferrin [13,18,19] or by direct assessment of serum Fe. Indirect assessment of Fe through other markers may have certain limitations in athletes [20]. For example, ferritin levels could only indicate the magnitude of Fe reserves and not its functional reserve [21]. Detection of Fe deficiency based on ferritin assessment is limited in athletes as physical training could induce inflammatory responses, especially in the acute phases, influencing ferritin concentrations [21]. Likewise, hemoglobin assessment might not be adequate since a low hemoglobin level might be due to an expansion of plasma volume [22]. Due to the limitations, according to previous authors it seems necessary to develop and implement standard protocols for the evaluation and treatment of Fe deficiency in women athletes [22]. Regarding the direct assessment of serum Fe, it is known to have a high diurnal variability. Morning Fe values are higher compared to values obtained in the afternoon [23]. Therefore, it could be an unreliable measure. Due to the different limitations of previous markers of Fe status, previous authors recommended using different markers and compartments to obtain a complete Fe status assessment [24]. 

Studies that have analyzed Fe status during the sports season, or at a specific time of the season, have used indirect hematological markers (erythrocytes, hemoglobin, and ferritin) [8,9,25]. However, to the best of our knowledge, no studies have been found analyzing hematological markers of Fe status together with Fe concentrations in various compartments. Therefore, the objectives of the present study were (i) to analyze changes in hematological parameters of Fe status and (ii) to analyze changes in Fe concentrations in different biological matrices (serum, plasma, urine, erythrocytes, and platelets) in women’s soccer players during a sports season.

## 2. Materials and Methods

### 2.1. Participants

Twenty-four semi-professional women’s soccer players from a Spanish second-division team participated in the study. The characteristics of the team are shown in Table 1. All participants trained and played home matches in the city of Caceres (Spain). The participants were informed of the objectives of the study by means of a signed informed consent form. The protocol detailed below was approved by the Biomedical Ethics Committee of the University of Extremadura (code 135/2020). The technical staff provided information on the equipment.

To participate in the study, the women’s soccer players had to meet the following inclusion criteria: (i) being resident in the same city one month before and during the study; (ii) not suffering from any type of chronic disease; (iii) not taking medication or supplementation that included EMT during the study period or the month prior to the first evaluation; (iv) not smoking or consuming drugs; (v) more than 5 years’ experience competing in soccer; (vi) not modifying nutritional and physical activity habits during the study; (vii) not going more than 30 days without training with the team; (viii) having regular menstrual cycles for at least six months prior to the start of the study and during the study; (ix) not suffering from problems related to the menstrual cycle; and (x) not using hormonal contraceptive methods.

Days before the start of the study, the participants completed a questionnaire to report on the characteristics of their menstrual cycle [26]. The questionnaire included general menstrual cycle questions (cycle length, duration of bleeding, age of onset, regularity, and pain). A researcher was available to assist the participants (Table 2).

### 2.2. Study Design

The study design was similar to that reported by Toro-Román et al. [26]. The duration of the study was eleven months, and three assessments were performed: assessment 1 (first week of training (August)), assessment 2 (mid-season, end of the first regular round (January)) and assessment 3 (last week of training (May–June)). In each assessment, nutritional intake was evaluated and hematological parameters, female hormones and Fe concentrations in plasma, serum, urine, erythrocytes, and platelets were determined. All assessments were performed in the same week of each month, in the morning at approximately the same time and in the same order. At the beginning of the study, to characterize the samples, anthropometry, body composition and physical condition were evaluated.

### 2.3. Nutritional Intake

Three days before each assessment, the participants completed a nutritional questionnaire. A document was provided indicating the amount and frequency of food intake during the three days prior to the assessments. The nutritional composition of each food was evaluated [27] and a conversion was carried out to estimate consumption [26]. Intake of energy, macronutrients, B12, folic acid and Fe were evaluated.

### 2.4. Blood and Urine Sample Collection

The techniques for obtaining blood and urine samples were similar to those reported by Toro-Román et al. [26] and Grijota et al. [24].

The participants were summoned at 8:00 a.m. in a fasting state. The players came with the first urine of the morning collected in 9 mL BD Vacutainer^®^ (Franklin Lakes, NJ, USA) tubes which were frozen at −80 °C until analysis.

Regarding blood samples, 15 mL was drawn using a 20 mL plastic syringe (Injekt, Braun, Melgunsen, Germany) and a sterile needle (Mirage Pic Solution, Trieste, Italy). Of the total, 5 mL were collected in Vacutainer^®^ tubes (Franklin Lakes, NJ, USA) with clot activator to determine hematological and hormonal parameters. The remaining 10 mL were extracted to determine Fe in different biological compartments. 

For plasma, 5 mL of blood were collected in 4 mL BD Vacutainer^®^ tubes with sodium citrate and centrifuged at 1800 rpm for 8 min. After centrifugation, platelet-rich plasma and erythrocytes were separated. The platelet-rich plasma was transferred into a 4 mL BD Vacutainer^®^ tube without additives and centrifuged at 3000 rpm for 10 min to separate the plasma from the platelets. The plasma was then transferred into 1.5 mL Eppendorf tubes and allowed to stand in the freezer until analysis. Then, 1 mL of pure water was added to the platelets adhering to the bottom of the tube after plasma collection, and they were vortexed (Cole-Parmer™, Stuart™, Vernon Hills, IL, USA) for dilution. After dilution, the contents were transferred to Eppendorf tubes and stored cold. Finally, the erythrocytes remaining after the first centrifugation were washed twice with 1 mL of 0.9% sodium chloride. After that, they were also collected in Eppendorf tubes and stored at −80 °C until analysis. 

### 2.5. Determination of Hematological Parameters of Iron Status, Female Hormones and Serum Iron

Hematological parameters of Fe status and serum Fe were determined using spectrophotometric techniques (Coulter Electronics LTD, Model CPA; Northwell Drive, Luton, UK). Female hormones were determined by ELISA (enzyme-linked immunosorbent assay), also with a spectrophotometer. The determination was performed by an external clinical analysis laboratory.

### 2.6. Determination of Fe in Plasma, Urine, Erythrocytes and Platelets

The technique was similar to that reported by Grijota et al. [24]. The method was developed entirely by the research support service of the University of Extremadura using inductively coupled plasma mass spectrometry (ICP-MS) (7900; Agilent Tech., Santa Clara, CA, USA). The linearity of the calibration curves for indium in plasma, serum, urine, erythrocytes, and platelets was greater than 0.985. The equipment was calibrated with several standards prepared from commercial multi element solutions of certified standards.

For plasma, serum and urine samples, the reagents used were nitric acid (HNO_3_) 69% Trace select from Fluka and ultrapure water obtained from a Milli-Q system manufactured by Millipore (Burlington, MA, USA). A Rhodium solution of 400 μgL^−1^ was used as internal standard.

For erythrocyte and platelet samples, the reagents used were 69% HNO_3_, hydrogen peroxide, both from Fluka’s Trace Select and ultrapure water obtained from a Milli-Q system manufactured by Millipore (USA). A 400 μg/L Yttrium and Rhodium solution was used as internal standard. 

The limits of detection (LOD) and limits of quantification (LOQ) of Fe in the different matrices throughout the investigation were (in μg/L): plasma and serum (LOD = 0.706; LOQ = 7.06), urine (LOD = 0.630; LOQ = 6.30), erythrocytes (LOD = 0.100; LOQ = 1.00) and platelets (LOD = 0.190; LOQ = 1.90). 

### 2.7. Anthropometry, Body Composition and Physical Fitness Tests

In the first evaluation, after blood samples were drawn and a free breakfast was eaten, anthropometry, body composition and physical condition were assessed (Table 3). The protocol was similar to the study by Toro-Román et al. [26].

Height, body weight and skinfolds (abdominal, suprascapular, subscapular, tricipital, thigh and calf) were evaluated. A wall stadiometer (Seca 220. Hamburg, Germany), a balance (Seca 769. Hamburg, Germany) and a Holtain© 610ND skinfold compass (Holtain© 610ND) (Holtain© Crymych, UK) were used. Assessments were performed following the guidelines of the Spanish Group of Kinanthropometry [28]. Fat percentage was estimated using the Yuhasz formula [29].

Before the physical fitness tests, a general warm-up consisting of hip and knee joint mobility followed by isometric squats was performed. Afterwards, participants ran for 10 min at 7 km/h.

Vertical jump tests were performed using an infrared platform (Optojump, Mycrogate, Mahopac, NY, USA). Participants performed two types of vertical jump: the squat jump (SJ) and countermovement jump (CMJ). For the SJ, participants initiated the movement from a position with their knees at a 90° angle and their arms resting on their hips. They then performed the vertical jump at the highest possible intensity. For the CMJ, participants began the execution from an upright position with their hands resting on their hips. Subjects performed a knee flexion-extension followed by a jump at maximum intensity. In both jumps, two attempts were performed with 30 s rest between jumps. The best jump was chosen for analysis.

To assess maximal aerobic capacity, a maximal incremental test was performed on a treadmill (Ergofit Trac Alpin 4000, Pirmasens, Germany), equipped with a gas analyzer (Geratherm Respiratory GMBH, Ergostik, Ref 40.400, Corp, Bad Kissingen, Germany). Participants ran in 1 min stages until exhaustion. The test started at 7 km/h and increased by 1 km/h every minute at a constant gradient of 1%. The above values were carried out in assessment 1 and used as descriptive values for the sample. 

### 2.8. Statistical Analysis

A value of *p* < 0.05 and a value of *p* < 0.01 were considered significant and highly significant differences, respectively. The IBM SPSS 25.0 Statistics program (IBM Corp., Armonk, NY, USA) was used. A one-way ANOVA was used to determine differences during assessment. The Bonferroni post hoc test was used to determine specific differences. The F value was also determined. Figures were created using GraphPad Software 8 Inc. (Boston, MA, USA). Results are expressed as mean ± standard deviation.

## 3. Results

Table 4 shows the results of the nutritional intake of the research participants. No significant differences were observed in any of the parameters analyzed.

Table 5 shows the evolution of progesterone and estradiol-17β concentrations throughout the study. There were no significant differences.

Table 6 presents the evolution of hematological parameters of Fe status throughout the sports season. There were significant differences throughout the study in erythrocyte, hemoglobin and mean corpuscular hemoglobin (MCH) values (*p* < 0.05). 

Specifically, there were differences in erythrocyte concentrations between assessment 1 and assessment 2 (*p* < 0.01), hemoglobin between assessments 1 and 3 (*p* < 0.01) and assessments 2 and 3 (*p* < 0.01), and MCH between assessments 1 and 2 (*p* < 0.01) and assessments 1 and 3 (*p* < 0.01).

**Figure 1:**
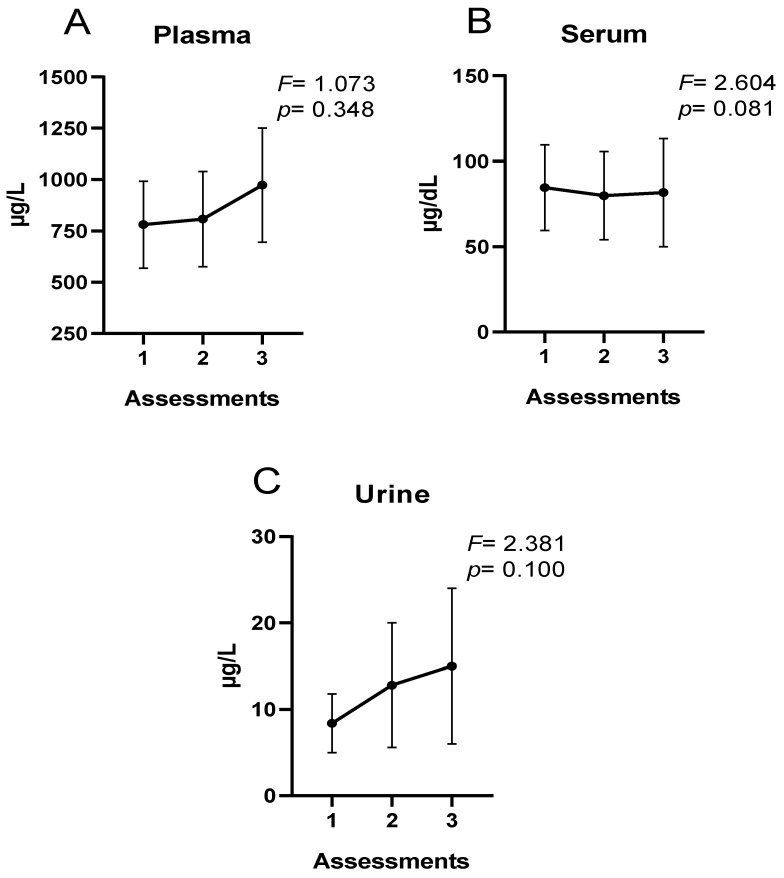
Extracellular Fe concentrations: (**A**) plasma concentrations; (**B**) serum concentrations; (**C**) urinary concentrations.

**Figure 2:**
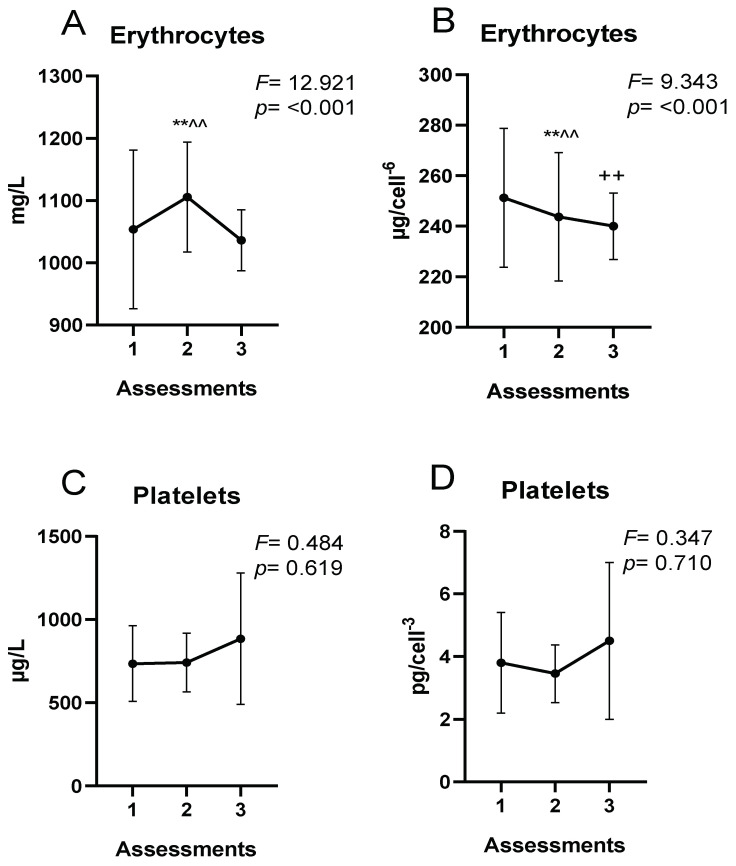
Intracellular concentrations of Fe. (**A**) Absolute erythrocyte concentrations; (**B**) concentrations relative to the number of erythrocytes; (**C**) absolute platelet concentrations; (**D**) concentrations relative to the number of platelets; ** *p* < 0.01 assessment 1 vs. assessment 2; ++ *p* < 0.01 assessment 1 vs. assessment 3; ^^ *p* < 0.01 assessment 2 vs. assessment 3.

Figure 1 and Figure 2 show the extracellular and intracellular Fe concentrations. There were no significant differences in extracellular Fe concentrations throughout the season (Figure 1). However, there were significant differences in erythrocyte Fe concentrations, being lower at the end of the season compared to the initial values (*p* < 0.01) (Figure 2).

Figure 1 shows the extracellular Fe concentrations during the sports season.

Figure 2 shows the erythrocyte and platelet Fe concentrations during the sports season.

## 4. Discussion

The objectives of the research study were (i) to analyze changes in hematological parameters of Fe status and (ii) to analyze changes in Fe concentrations in different biological matrices (serum, plasma, urine, erythrocytes and platelets) in women’s soccer players during one season. Different hematological parameters have been used to analyze Fe status in women’s team sports [8,30,31]. However, to the best of our knowledge, this is the first study to evaluate Fe concentrations in different compartments (plasma, serum, urine, erythrocytes and platelets) together with hematological parameters of Fe status. The Fe concentrations obtained in each compartment, by ICP-MS, were within the ranges reported in other investigations with similar techniques [32,33,34].

Fe is an essential mineral for numerous processes such as oxygen transport and energy production [35]. Women athletes tend to experience a higher incidence of Fe deficiency [36], possibly as a result of increased demand to compensate for menstruation [8]. Low energy intake, vegetarian diets, and endurance exercise have also been proposed as factors affecting Fe stores [36]. In relation to the above, it is important that an athlete’s Fe status is routinely monitored, and that appropriate action is taken if correction of a deficiency is required. 

Monitoring the menstrual cycle is important in studies assessing Fe status because fluctuations in female sex hormones during the menstrual cycle could influence hematologic markers of Fe status [37]. Previous studies found reduced levels of serum Fe, ferritin, during the follicular phase compared to other phases of the menstrual cycle [38,39]. Therefore, in the present investigation, the characteristics of the menstrual cycle of the participants were qualitatively studied and two female hormones were determined, with no significant changes throughout the investigation. Like Fe, other mineral elements could vary their concentrations throughout the menstrual cycle [40]. Therefore, based on the results, the assessments were performed at approximately the same phase of the menstrual cycle.

Fe is the most abundant mineral element in the body [41]. When the intake and reserve of Fe is deficient, physical and cognitive performance decreases [42]. Individuals do not have mechanisms to restore Fe losses. Therefore, adequate dietary intake is essential for athletes during periods of intense training [43]. Fe intake was higher than the reference dietary intake (9–11 mg/day) [44]. Women tend to have lower total dietary intake and, in turn, lower Fe intake compared to men [45]. Previous authors reported in 16 international women’s soccer players a mean Fe intake of 12.1 mg/day [46]. On the other hand, mean Fe intakes of 8.8 mg/day were reported in 41 professional Polish women’s soccer players [47]. Therefore, the Fe intake in the present study was higher than reported studies. 

Soccer players exposed to a demanding schedule during a competitive period may be predisposed to Fe deficiency that could compromise their performance and metabolic health toward the end of the season [48]. Accumulated fatigue and inadequate recovery time during a competitive period may predispose soccer players to alterations in Fe status [10].

Athletes often have out-of-range hematological parameters due to various factors such as regular physical training, physiological and psychological stress, or environmental conditions, among others [49]. A wide variety of physiological markers have been used for long-term monitoring of athletes [3]. In particular, hematological markers have been considered important indicators of the body’s adaptation in response to different training loads [50]. In the present study, different hematological parameters related to Fe status were analyzed. The hematological values reported in the present study were lower than those reported in 28 first- and second-division women players [51] and in 25 elite Korean women players [25]. In men, no significant changes in hemoglobin, hematocrit, and mean corpuscular volume (MCV) concentrations were observed over a season [52]. However, there was an increase in erythrocyte concentrations compared to baseline values. In men’s soccer players, after 45 days of training, there were decreases in hemoglobin and hematocrit values [53]. Regarding ferritin and transferrin values, Gropper et al. [54] reported lower concentrations in 17 university women’s soccer players compared to the present study. On the other hand, Fallon et al. [55] reported higher ferritin and transferrin concentrations in Australian women’s soccer players compared to the present study. 

Normally, endurance training causes a decrease in erythrocytes, hemoglobin, and hematocrit [56]. This could be due in part to the expansion of plasma volume [57] resulting from an increase in aldosterone production accompanied by osmotically active plasma proteins. Although not analyzed in the present investigation, previous authors have observed increases in plasma volume along with a decrease in hemoglobin and hematocrit in soccer players [53], similar to what occurred in the first two assessments in the present study. Additionally, it is known that physical exercise causes an increase in the rate of hemolysis as a result of trauma, oxidative damage due to elevated superoxide production, or osmotic changes that induce changes in red blood cell volume, increasing their fragility [14]. Exercise-induced hemolysis has been associated with exercise intensity [58]. During activities involving running or jumping, red blood cells in the capillaries of the sole of the foot are destroyed by the mechanical forces experienced upon impact with the ground. These mechanisms may explain why both exercise duration and intensity are negatively associated with hemoglobin, hematocrit, and serum ferritin concentrations in highly trained athletes [7]. In relation to the above, Peeling et al. [59] reported a considerable influence of physical training on hemolysis, stating that frequent hemolytic episodes induced by physical training could negatively influence Fe stores.

Regarding extracellular Fe concentrations, no differences were observed throughout the season in the present study. However, the trend in plasma and urine was incremental, whereas in serum, Fe concentrations were stable. In urine, increases in Fe elimination have been observed after strenuous exercise in normothermic and hyperthermic environments [60]. On the other hand, during 4 days of light physical exercise in older people, increases in the percentage of people with urinary Fe losses were observed [61]. In cross-country runners during 2 months of training, mild urinary Fe losses were reported with no increase in Fe-deficient runners [11]. Regarding plasma concentrations, Mettler and Zimmermann [62] reported elevated plasma Fe values in active individuals and marathon runners. On the other hand, Ponorac et al. [63] and Sandström et al. [64] reported lower Fe concentrations in women athletes compared to a control group.

The possible Increases in plasma and urinary Fe could be multifactorial. Hemolysis occurring during training, specifically in soccer, could be an important factor [65,66]. The predominant actions in soccer (jumping, sprinting, changes in direction, kicking, etc.) could increase muscle damage and hemolysis. Elevated body temperature and metabolic acidosis reduce the osmotic resistance of erythrocytes. Structural alterations of erythrocyte membranes increase the susceptibility of these cells to hemolysis, leading to elevated plasma levels of free Fe [65,66]. On the other hand, increases in urinary Fe may be related to increases in hematuria levels. Hematuria may result from trauma to the bladder wall during physical exercise. Fe may also be excreted in the urine due to increased rapid intravascular hemolysis [67]. Bladder trauma in runners and intravascular hemolysis, especially in athletes subjected to capillary trauma [68,69], have been implicated as factors in urinary hemoglobin loss.

As for intracellular Fe concentrations, the reduction of erythrocyte Fe at the end of the season could be due to the hemolysis produced during the regular physical training throughout the season discussed above. The half-life of erythrocytes in athletes could be significantly shorter than in physically inactive subjects [70]. Exercise-induced hemolysis could be implicated in the suboptimal erythrocyte Fe status of athletes [71]. This could trigger Fe loss as a consequence of erythrocyte membrane destruction and subsequent release of hemoglobin and Fe to extracellular compartments [43]. The rate of erythrocyte destruction could be altered as a consequence of repetitive physical training [72]. Regular physical exercise causes stressful physiological situations, such as increased oxidative stress, which may alter erythrocyte membrane properties [70].

Finally, the present study is not without limitations: (i) plasma volume was not evaluated. During physical exercise, losses of body water can occur through sweating, which can induce hemoconcentration; (ii) the sample size was not calculated to be able to understand the magnitude of the sample obtained; (iii) the technical error of measurement in the physical condition and anthropometric assessments was not calculated; (iv) the sample was small; and (v) other indirect markers of Fe status such as transferrin saturation or total iron binding capacity (TIBC) were not assessed.

## 5. Conclusions

During a regular sports season, changes occur in the hematological parameters related to Fe status and intracellular Fe concentrations in women’s soccer players.

Hemoglobin and MCH increase at the end of the season in relation to the initial values. The number of erythrocytes decreases in the middle of the season and is restored at the end of the competitive period. Erythrocyte Fe concentrations decreased at the end of the season compared to initial values. Erythrocyte Fe reduction may increase the extracellular Fe concentration.

Monitoring hematological parameters of Fe status and total Fe concentrations is essential to identifying a healthy and optimal performance status. Assessment of Fe status should not be limited to hematological markers. Moreover, the assessment of total Fe concentrations should not be limited to plasma or serum because significant changes can occur intracellular level.

## Figures and Tables

**Table 1:** Participants and training characteristics.

	Women’s Soccer Players
N	24
Age (years)	23.37 ± 3.95
Experience (years)	14.51 ± 4.94
Training (weeks)	39
Training sessions (n)	133 ± 25
Training (min)	10,578 ± 3227
Matches played (n)	36
Injuries (nº)	8
Absence from training (days)	14 ± 10

**Table 2:** Menstrual cycle characteristics.

		Women’s Soccer Players
Age of first appearance (years)		13.5 ± 1.15
Regular menstruation (%)		100.00
Duration of bleeding (days)		4.77 ± 1.47
Amount of bleeding (%)	Light	11.11
	Moderate	77.77
	Heavy	11.11
Menstrual cycle (days)		27.93 ± 2.78
Cessation of menstruation (%)	Never	88.88
	Sometimes	12.22

**Table 3:** Anthropometry, body composition and physical fitness characteristics.

	Women’s Soccer Players
Height (m)	1.65 ± 0.06
Weight (kg)	59.58 ± 7.17
Σ6 skinfolds (mm)	94.62 ± 18.54
Fat (%)	18.16 ± 2.74
SJ (s)	0.539 ± 0.045
CMJ (s)	0.569 ± 0.055
Incremental test time (min)	9.18 ± 1.12
VO_2max_ (mL/min/kg)	39.72 ± 6.22
VO_2max_ (L/min)	2.28 ± 0.40

Σ: summatory; SJ: squat jump; CMJ: countermovement jump; VO_2max_: maximum oxygen uptake.

**Table 4:** Nutritional intake.

		Women’s Soccer Players	*F*	*p*
Energy (kcal/day)	Assessment 1	1578.1 ± 316.2	1.188	0.307
	Assessment 2	1681.5 ± 427.3		
	Assessment 3	1697.3 ± 386.1		
Proteins (g/day)	Assessment 1	90.4 ± 21.6	0.841	0.473
	Assessment 2	96.2 ± 18.3		
	Assessment 3	92.6 ± 20.4		
Lipids (g/day)	Assessment 1	48.3 ± 12.3	1.467	0.219
	Assessment 2	55.6 ± 15.3		
	Assessment 3	60.3 ± 20.6		
Carbohydrates (g/day)	Assessment 1	206.1 ± 81.3	1.956	0.156
	Assessment 2	241.5 ± 56.1		
	Assessment 3	235.8 ± 61.7		
Folic acid (µg/day)	Assessment 1	521.4 ± 61.8	1.145	0.391
	Assessment 2	511.6 ± 49.1		
	Assessment 3	500.8 ± 61.8		
B12 (µg/day)	Assessment 1	5.8 ± 1.7	0.378	0.550
	Assessment 2	6.1 ± 1.9		
	Assessment 3	6.4 ± 2.1		
Fe (mg/day)	Assessment 1	12.5 ± 2.5	0.247	0.713
	Assessment 2	12.6 ± 1.8		
	Assessment 3	12.7 ± 2.6		

Fe: iron.

**Table 5:** Concentration of female hormones.

		Women’s Soccer Players	*F*	*p*
Progesterone (ng/mL)	Assessment 1	2.65 ± 3.88	0.052	0.998
	Assessment 2	2.38 ± 3.21		
	Assessment 3	2.31 ± 2.89		
Estradiol-17β (pg/mL)	Assessment 1	74.04 ± 45.30	0.165	0.894
	Assessment 2	71.32 ± 39.25		
	Assessment 3	68.30 ± 40.93		

**Table 6:** Hematological parameters of Fe status.

		Women’s Soccer Players	*F*	*p*
Erythrocytes (millions)	Assessment 1	4.37 ± 0.22	3.767	0.028
	Assessment 2	4.19 ± 0.27 **		
	Assessment 3	4.35 ± 0.27		
Hemoglobin (gr%)	Assessment 1	12.79 ± 0.92	13.414	<0.001
	Assessment 2	12.29 ± 0.93		
	Assessment 3	13.82 ± 0.95 ++^^		
Hematocrit (%)	Assessment 1	36.34 ± 2.43	2.371	0.101
	Assessment 2	34.89 ± 2.61		
	Assessment 3	35.88 ± 2.43		
MCV (fL)	Assessment 1	87.01 ± 3.48	0.184	0.832
	Assessment 2	86.55 ± 3.22		
	Assessment 3	87.04 ± 3.17		
MCH (Pg)	Assessment 1	29.90 ± 1.39	12.507	<0.001
	Assessment 2	31.43 ± 1.45 **		
	Assessment 3	31.75 ± 1.38 ++		
Platelets (miles)	Assessment 1	196.00 ± 38.01	2.631	0.079
	Assessment 2	219.08 ± 34.19		
	Assessment 3	204.39 ± 31.52		
B12 (pg/mL)	Assessment 1	447.95 ± 137.16	0.249	0.780
	Assessment 2	443.28 ± 103.48		
	Assessment 3	466.34 ± 96.96		
Folic acid (ng/mL)	Assessment 1	5.24 ± 2.08	0.968	0.385
	Assessment 2	6.15 ± 2.16		
	Assessment 3	5.72 ± 1.67		
Ferritin (mcg/L)	Assessment 1	32.26 ± 15.31	1.452	0.241
	Assessment 2	21.62 ± 7.80		
	Assessment 3	28.00 ± 13.23		
Transferrin (mcg/L)	Assessment 1	270.82 ± 37.92	1.003	0.372
	Assessment 2	273.16 ± 36.60		
	Assessment 3	259.08 ± 26.93		

** *p* < 0.01 assessment 1 vs. assessment 2; ++ *p* < 0.01 assessment 1 vs. assessment 3; ^^ *p* < 0.01 assessment 2 vs. assessment 3; F: MCV: mean corpuscular volume; MCH: mean corpuscular hemoglobin.

## Data Availability

Not applicable.
